# Emotional experiences reported by psychologists attending patients with suicidal crises in a service in northeastern Brazil: A qualitative study

**DOI:** 10.1192/j.eurpsy.2021.1325

**Published:** 2021-08-13

**Authors:** E. Turato, L. Guerra, C.A. Oliveira, B. Gondinho, P.A. Leme

**Affiliations:** Medical Psychology And Psychiatry, University of Campinas, Campinas, Brazil

**Keywords:** mental health, Qualitative Research, Suicide Attempt, psychoterapy

## Abstract

**Introduction:**

Contextualization: Emotional experiences of psychologists related to the care of suicide crises are important since the health professional has been trained to save lives. It makes him apprehend the aggressive side of suicide and symbolize it as an attack. When the patient’s desire to liveceases, the professional may feel confused, since his/her profession/vocation was confronted.

**Objectives:**

AIM: To explore and interpret the meanings of emotional experiences reported by psychologists who care for patients in suicide crises.

**Methods:**

Strategies: clinical-qualitative design, semi-directed interviews with open-ended questions in-depth. Six clinical psychologists from a Brazilian city participated, with a sample closed by information saturation. Interviews audio recorded, full transcribed and categorized by Qualitative Content Analysis. Results were peer-reviewed in meetings in a Qualitative Research Study Group.

**Results:**

Findings: Three emerging categories - (1) Ambivalent emotions as challenges for clinical management, (2) The non-paralyzing experience of emotions, (3) The management that is learned in practice.

**Conclusions:**

Considerations: assistance to patients with a suicidal crisis can generate ambivalent emotions, not always paralyzing. When recognized and elaborated can assist in clinical practice. It can be tools that will support qualified approaches, especially in relation to suicide. As a public health problem, it demands a combination collective actions with effective individual clinical approaches.

